# Remaining Useful Life Prediction Based on Adaptive SHRINKAGE Processing and Temporal Convolutional Network

**DOI:** 10.3390/s22239088

**Published:** 2022-11-23

**Authors:** Haitao Wang, Jie Yang, Lichen Shi, Ruihua Wang

**Affiliations:** 1 School of Mechanical and Electrical Engineering, Xi’an University of Architecture and Technology, Xi’an 710055, China; 2 Institute of Electromechanical System Detection and Control, Xi’an University of Architecture and Technology, Xi’an 710055, China

**Keywords:** remaining useful life, deep learning, temporal convolutional network, adaptive shrinkage processing

## Abstract

The remaining useful life (RUL) prediction is important for improving the safety, supportability, maintainability, and reliability of modern industrial equipment. The traditional data-driven rolling bearing RUL prediction methods require a substantial amount of prior knowledge to extract degraded features. A large number of recurrent neural networks (RNNs) have been applied to RUL, but their shortcomings of long-term dependence and inability to remember long-term historical information can result in low RUL prediction accuracy. To address this limitation, this paper proposes an RUL prediction method based on adaptive shrinkage processing and a temporal convolutional network (TCN). In the proposed method, instead of performing the feature extraction to preprocess the original data, the multi-channel data are directly used as an input of a prediction network. In addition, an adaptive shrinkage processing sub-network is designed to allocate the parameters of the soft-thresholding function adaptively to reduce noise-related information amount while retaining useful features. Therefore, compared with the existing RUL prediction methods, the proposed method can more accurately describe RUL based on the original historical data. Through experiments on a PHM2012 rolling bearing data set, a XJTU-SY data set and comparison with different methods, the predicted mean absolute error (MAE) is reduced by 52% at most, and the root mean square error (RMSE) is reduced by 64% at most. The experimental results show that the proposed adaptive shrinkage processing method, combined with the TCN model, can predict the RUL accurately and has a high application value.

## 1. Introduction

With the rapid development of computing methods and information technology, modern production systems have become more complex [1]. In recent years, the prognosis and health management (PHM) has been a common and effective way to improve the work availability, safety, supportability, maintainability and reliability of modern industrial equipment and reduce life cycle costs, and thus has received widespread attention from both academia and the industry. Among the processes involved in the PHM framework, the remaining useful life (RUL) prediction represents a key task for PHM [2] and forms the basis for the decision-making of management activities. The purpose of the RUL prediction includes predicting when a system or component will operate normally, warning of impending failures, and helping to prevent industrial mishaps to a considerable extent. Therefore, an efficient RUL prediction method is urgently needed in the industrial field. Thus, the construction of an accurate RUL prediction model is essential to realizing the above-mentioned tasks.

In the past decade, RUL prediction technology has made great progress, mainly including model-based methods, data-driven methods and hybrid methods [3,4]. Among them, model-based methods usually need to establish failure degradation models for research objects, and generally do not have generalization. Due to the complexity of working conditions, the complexity of mechanical equipment and the different degradation mechanisms, the process of obtaining failure models is complex, and the prediction effect is difficult to guarantee [5]. The data-driven method is useful to explore the relationship with the remaining life from the data collected by sensors through machine learning and statistical methods [6]. Traditional data-driven methods (such as support vector machine [7], neural network [8], etc.) have achieved some results in residual life prediction. However, with the complexity and integration of mechanical equipment, the collected sensor data are becoming larger and larger and it is difficult to obtain the characteristic relationships contained therein, so there are certain errors in the accuracy of residual life prediction results.

Deep learning has a strong nonlinear mapping ability and feature extraction ability, and it is increasingly used in the field of RUL prediction and health monitoring [9]. In RUL prediction, recurrent neural network and its improved variants have been widely used. For example, Senanayake et al. [10] used an autoencoders and RNN to predict bearing RUL. Luo et al. [11] used a BiLSTM model to predict the degradation trend of roller bearing performance, and verified the effectiveness and robustness of the proposed method through experiments. Zhang et al. [12] proposed a novel bidirectional gated recurrent unit with a temporal self-attention mechanism (BiGRU-TSAM) to predict RUL. Zhang et al. [13] proposed a dual-task network based on a bidirectional gated recurrent unit (Bi-GRU) and a multi-gate mixture of experts (MMoE), which can simultaneously evaluate the health status and predict the RUL of mechanical equipment. These methods solve the difficult problem of unpredictable RUL under specific conditions. However, RNN and its variants can capture potential time patterns based on cyclic recursive structure, but it is difficult to design and train due to its complex internal structure. In addition, the problem of gradient explosion and gradient disappearance often leads to the low accuracy of RNN training [14]. The emergence of convolutional neural networks (CNN) makes the prediction method of time series data no longer limited to RNNs [15]. CNN has the advantage of parallel computing, and when the receptive field increases, the network model can obtain more historical information; therefore, it has also been widely used and has achieved very good results. For example, in Ge et al. [16], a short-term traffic speed prediction method based on graph attention convolution network was proposed, and good prediction results were obtained. Li et al. [17] proposed a CNN-based RUL prediction method trained with a cycle-consistent learning scheme to align the data of different entities in similar degradation levels. Lin et al. [18] proposed a trend attention fully convolution network (TaFCN) to further improve the prediction performance. However, when CNN processes long time series, it often needs a deeper structure to obtain enough receptive fields, which will reduce the training efficiency.

The temporal convolutional networks (TCNs) have been the latest improvement in the CNN structure, which extracts historical data using the dilated causal convolution (DCC). The dilated causal convolution usually includes fewer layers than the classical CNN but can capture the same receptive field. Therefore, TCNs have a better time series prediction ability than CNNs [19]. In addition, TCN has no cyclic connection, which makes it more efficient in training than RNN in computation. Recent studies have pointed out the potential of TCN in prediction; for instance, Sun et al. [20] used a TCN to predict the RUL of rotating machinery and Gan et al. [21] used a TCN to predict the wind speed range of wind turbines successfully. However, the vibration signal we collected from the sensor contains noise. In RUL prediction, TCN is often affected by noise when extracting degradation features, which makes it impossible to accurately capture degradation features from historical data, leading to low prediction accuracy. Moreover, due to the impact of changes in working environment and load, the noise intensity will vary with time. The question of how to adaptively solve the redundant information such as noise is particularly important for RUL prediction.

To solve these problems, this paper proposes a RUL prediction method based on adaptive shrinkage processing and temporal convolution network (AS-TCN). Firstly, the vibration signals monitored by multiple channels are directly used as the input of the prediction network, without prior knowledge to extract features. Secondly, in AS-TCN, TCN residual connection and dilated causal convolution are used to extract long-term historical information, and an adaptive shrinkage processing sub-network is introduced to adaptively eliminate different noises. Finally, the PHM2012 bearing data set and XJTU-SY bearing data set are compared with the three most advanced methods, respectively. The average MAE is reduced by 52% at most, and the average RMSE is reduced by 64%, which verifies the effectiveness of the proposed method.

The main contributions of this paper can be summarized as follows:

(1) A new framework of RUL prediction based on AS-TCN is proposed. It can directly use multi-channel monitored data as network input, without prior knowledge to extract features and effectively capture the key degradation information of bearings, thus realizing the end-to-end prediction process.

(2) Using a TCN network to build the main network can help it remember a large amount of complete historical information, avoid the shortcomings of long-term dependence and improve the accuracy of RUL prediction.

(3) Add an adaptive shrinkage processing subnet to the TCN block. The sub-network can adaptively adjust the threshold of the soft threshold function to minimize the redundant information related to noise while retaining the features that can better reflect the degradation information.

The rest of this article is organized as follows. Section 2 briefly introduces the theoretical background of the TCN and adaptive contraction mechanism. Section 3 describes the AS-TCN internal structure and implementation process. Section 4 verifies the RUL predictive performance of the proposed rolling bearing method. Finally, Section 5 concludes the paper and presents future work directions.

## 2. Theoretical Overview of TCN

### 2.1. Dilated Causal Convolution (DCC)

Causal convolution was first used in the WaveNets model [22] to learn the input audio data before time τ to predict the output at time (τ+1). The output at time τ can be obtained only from the input data before time τ; namely, the prediction model at time τ cannot rely on any future time step. Causal convolution adopts the method of unilateral filling and ensures that the input size is consistent with the output size by performing the zero filling on the input data and thus avoiding the leakage of information that never came to the past [23]. 

Since there is no recurrent connection in casual convolution, a parallel input of time series data can be adopted, so causal convolution has a faster training speed than RNNs, particularly for the large sample time series [24]. However, when dealing with long sequence data, causal convolution requires a deeper network structure or a large convolution kernel to enhance the receptive field of neurons in a neural network. For this reason, the TCNs have been combined with the DCC technology. The dilated causal convolution has been achieved by introducing the dilated convolution into the causal convolution so as to increase the receptive field, which can be expressed as follows:

(1) $$F\left(s\right)=\sum_{i=0}^{k-1}f\left(i\right)\cdot x_{{}_{s-d\cdot i}}$$

where κ is the size of the convolution kernel, d is the dilation factor, $\left(\cdot \right)$ represents the convolution calculation and subscript s−d·i indicates the past direction.

**Figure 1:**
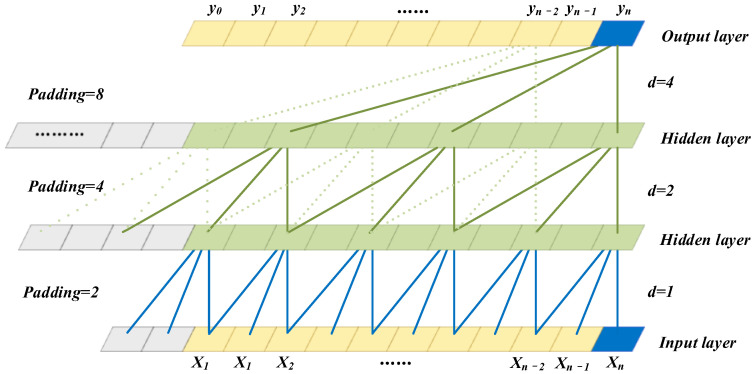
The DCC architecture.

The DCC architecture with a kernel size of κ=3 is presented in Figure 1. In the first hidden layer, the dilation factor is one, indicating that one neuron is omitted between the selected set of neurons. In the second hidden layer, with a dilation factor of two, three neurons are omitted between selected neurons. Each layer of TCN is a residual block, and the dilation factor of the convolutional neurons in each residual block increases at a rate of ($2^{n}-1$) from shallow to deep, ensuring a good performance of memorizing historical information.

### 2.2. Residual Module

The TCN uses residual learning to simplify deep network training. He et al. [25] proposed the residual modules for the first time and achieved promising results in speech recognition [26] and image processing [27]. The core idea has been to introduce a skip connection operation that skips one or more layers. Generally, residual learning refers to the process of undirect usage of a superimposed nonlinear layer to achieve the actual mapping;$\;H\left(X\right)$ the stacked nonlinear layers fit the residual map $F\left(X\right)$, and the original, required mapping of $H\left(X\right)$ is modified to $F\left(X\right)+X$. In the residual connection, an identity skip connection that bypasses the residual layer is introduced. By establishing a cross-layer connection between two layers that are apart, multiplexing of the output characteristic map of a convolution layer is enhanced, which improves network performance. At the same time, the problem of gradient dissipation or gradient explosion caused by a large number of layers can be effectively avoided. The residual is mapped as follows:

(2) $$F\left(X\right)=H\left(X\right)-X$$

**Figure 2:**
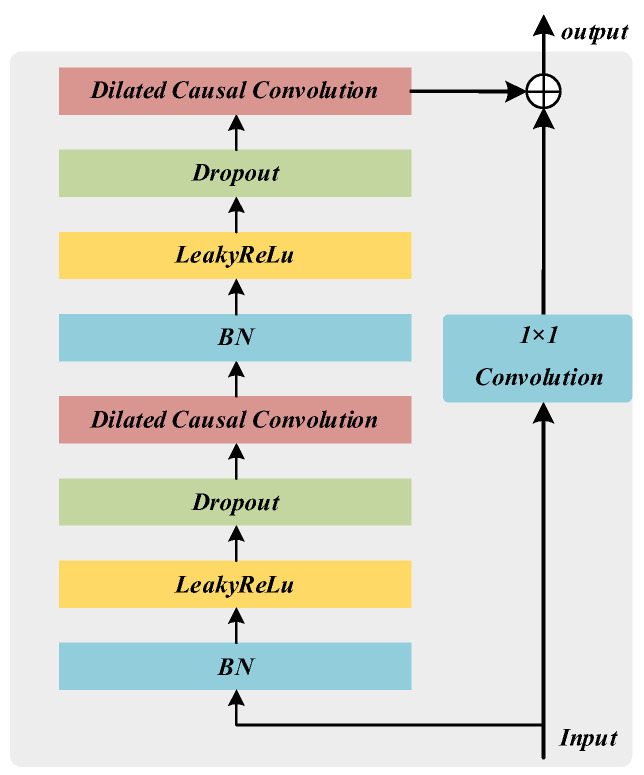
The block diagram of the fully pre-activated residual module.

Batch normalization (BN) [28] has usually been required for deep network training. The BN uses the mean and standard deviation of small batches to adjust the intermediate output of a network, which improves the stability of the intermediate output and minimizes overfitting. The activation function and BN have usually been added after the convolution operation in the conventional CNN structure. Many studies on primitive residual networks have analyzed how the combination of activation function and BN at different locations affects the network performance [29]. The results have shown that the fully pre-activated structure is superior to other structures in reducing overfitting and improving the generalization ability of the network. Therefore, this study aims to achieve full pre-activation of the residual connection by adding the activation function and BN before the dilated causal convolution. The improved residual structure is presented in Figure 2.

### 2.3. Adaptive Shrinkage Processing

**Figure 3:**
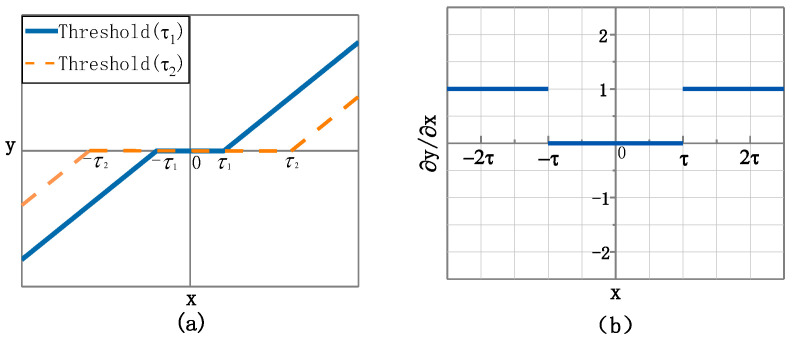
Soft-thresholding function. (**a**) Soft threshold functions for different thresholds. (**b**) The derivative of the soft threshold function.

**Figure 4:**
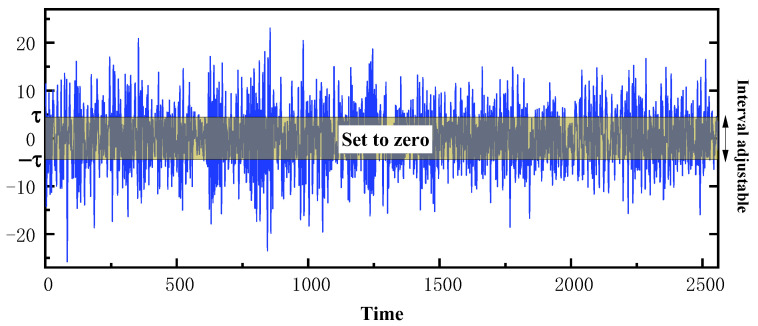
The shrinkage processing results.

Shrinkage processing refers to soft thresholding, which is a function that shrinks input data in the direction of zero to retain negative or positive characteristics and sets the characteristics approaching zero to zeros. In this way, it has been proven that useful information can be well preserved and noise related features can be eliminated [30]. The soft-thresholding function is given by Equation (3), where y and x are the output and input features, respectively, as shown in Figure 3a; they are two soft threshold functions with different thresholds. The boundary in the soft-thresholding function is controlled by a threshold τ, whose value in the interval of $\left[-\tau ,\tau \right]$ is set to zero. The soft-thresholding function can adjust the threshold value τ to shrink, as shown in Figure 4. Meanwhile, the soft-thresholding function’s derivative is defined by Equation (4) and presented in Figure 3b, where it can be seen that the derivative of the soft-thresholding function is either zero or one. Therefore, using the soft-thresholding function is beneficial to prevent gradient and exploding.

(3) $$soft\left(x,\tau \right)=y=\left\{\begin{array}{c} x+\tau ,x<-\tau \\ 0,\left|x\right|\leq \tau \\ x-\tau ,x>\tau \end{array}\right.$$

(4) $$\frac{\delta y}{\delta x}=\left\{\begin{array}{c} 1,x<-\tau \\ 0,\left|x\right|\leq \tau \\ 1,x>\tau \end{array}\right.$$

To detect the degradation information of equipment comprehensively, this study uses the operation-to-failure data collected by different sensors as a network training dataset. However, due to environmental impact, changes in operating conditions, performance degradation and other factors, the noise level also changes. Therefore, when the signal samples are converted into the feature map through the stack layer, it is necessary to customize the threshold of the feature map. To this end, an adaptive shrinkage training subnet is constructed in the TCN framework. This subnet adaptively adjusts threshold τ during training by optimizing operations with the purpose of minimizing deviations from basic facts and model output.

**Figure 5:**
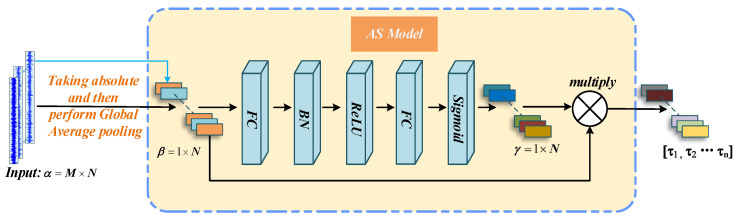
The block diagram of the adaptive shrink processing mechanism.

The working mechanism of the adaptive shrinkage processing is illustrated in Figure 5. Suppose that the input tensor is α, having M rows and N columns; namely, N characteristic graphs are used to calculate the absolute value of the input layer tensor one by one, and then the average value of each column is calculated in the global average pooling layer; the average value has one row and N matrices, and it is represented by β and processed by the full connection (FC) layer and the BN layer in turn. The activation function of the last FC layer is set to the “Sigmoid” function so that the shape invariant γ is in the range of$\;\left[0,1\right]$. Through the process of multiplying β and γ by elements, each feature map has its own threshold vector $\left[\tau_{1},\tau_{2},\ldots \tau_{n}\right]$, as the result of adaptive shrinkage. Finally, the input feature and soft threshold vector realize shrinkage processing via Equation (3). 

### 2.4. AS-TCN Block

**Figure 6:**
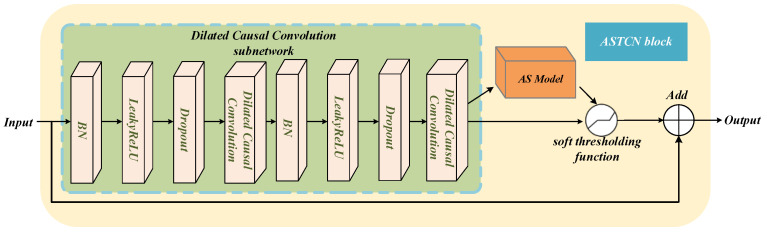
The AS-TCN block structure.

The AS-TCN block consists of DCC and an adaptive shrinking sub-network. After a series of operations, the input data, which represent a picture with redundant information and degradation features, are input to the TCN block, and different thresholds are obtained by the adaptive shrinkage subnetwork for different features. Next, the soft threshold function is used to eliminate redundant information to retain degenerate features. Moreover, the TCN block uses an identity path to reduce the difficulty in model training. The internal details of the DCC sub-network stacked with custom layers are presented in Figure 6. The LeakyRELU activation function is used at the cost of gradient sparsity, so the module is more robust in terms of optimization [31].

## 3. AS-TCN-Based RUL Prediction Method

### 3.1. RUL Prediction Process

**Figure 7:**
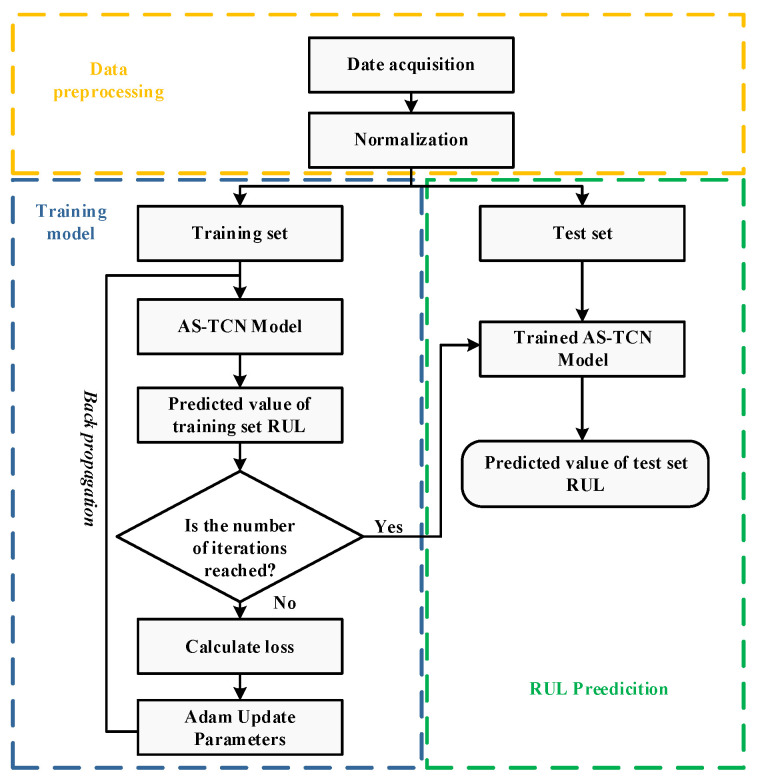
The AS-TCN network prediction process.

The RUL prediction process is illustrated in Figure 7, where it can be seen that data collection is performed first, and then sensor signals from different channels are collected. Next, the signals are standardized, and the preprocessed data are divided into test and training sets. Then, the training set is used to train the network through a predefined number of iterations. During the model training, the back-propagation method is used; the Adam optimizer is employed to reduce the loss function value (loss), and the optimal structural parameters are determined. After 200 iterations, the trained network model is obtained.

The trained network model is used to perform RUL prediction on the test set. The simulation results are expressed according to the fit between the RUL predicted value curve of the test set and the true value curve, and the absolute error (MAE) and mean square root error (RMSE) are used as indicators to evaluate the prediction effect.

### 3.2. AS-TCN Prediction Model

**Figure 8:**
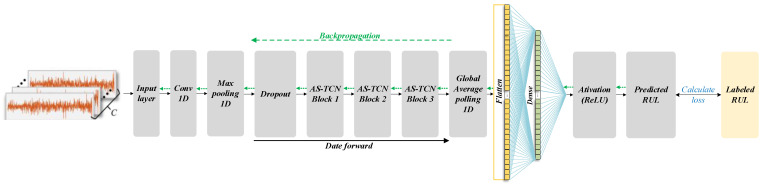
The AS-TCN network prediction model.

The structure of the RUL prediction network based on the AS-TCN designed in this paper is shown in Figure 8. The sensor data collected from the two channels, namely the vibration signals in the x and y directions, are used as the network input. The two-dimensional input tensor passes through the one-dimensional roll-up layer, the maxpool layer, the dropout layer, three stacked TCN modules, the Global-Average-pool block and a full connection layer. The dropout layer is used to reduce overfitting, compare the network output with the actual RUL value and back-propagate the error.

Network parameters include trainable parameters (such as the weights and offsets of convolution kernels) and untrained super parameters (such as the number and size of convolution kernels). The super parameter needs to be set in advance, and its influence on the network prediction effect and training time needs to be comprehensively considered. After repeated experiments and comparisons, the final super parameter setting is shown in Table 1. The Adam algorithm is selected as the optimizer during training, with a learning rate of 0.001, and a total of 200 times of training.

## 4. Experimental Verification

### 4.1. Case Study 1: PHM2012 Bearing Dataset

#### 4.1.1. Dataset Introduction

**Figure 9:**
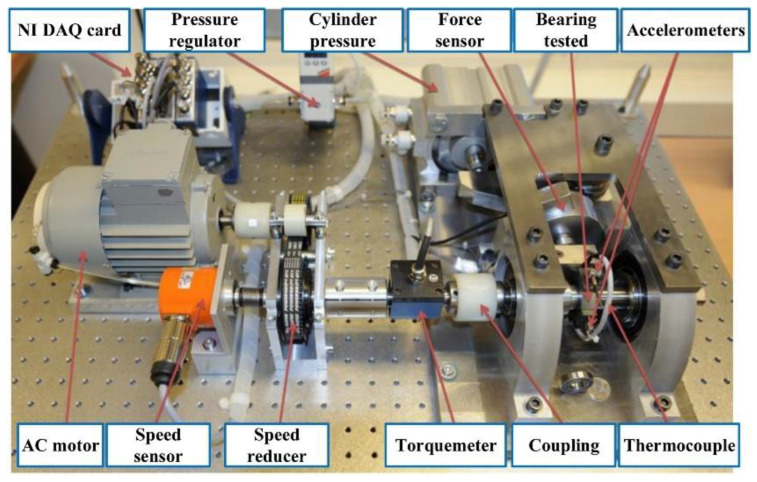
The PRO-NOSTIA test bench.

**Figure 10:**
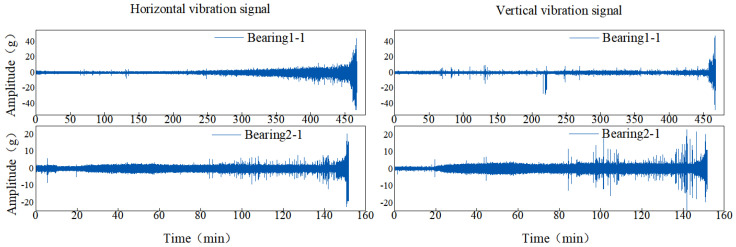
The full-life data of bearings 1-1, 2-1.

The proposed prognostic method was validated using two accelerated rolling bearing degradation test datasets. The bearing operation-to-failure dataset [32] released by the IEEE PHM2012 Data Challenge was measured by the PRO-NOSTIA test rig, as shown in Figure 9. The data were collected by two acceleration sensors in the horizontal and vertical directions, separately; the sampling frequency was 25.6 kHz, and the data were recorded every 10 s for 0.1 s, so the vibration data for each sampling included 2560 points. For safety reasons, the experiment was stopped when the amplitude of the vibration data exceeded 20 g. The measured bearing failure time was defined as the time when the amplitude was greater than 20 g. The proposed prediction model was verified by using the operation-to-failure data under conditions one and two, as shown in Table 2. The life cycle data of bearings 1-1 and 2-1 are shown in Figure 10.

The server configuration used in the laboratory was as follows: the processor was an Intel^®^ Xeon E5 2696 v2 @ 2.5 GHz, the memory was 128 GB and the GPU was a Nvidia^®^ GeForce 3070Ti (8 GB); the operating system was Microsoft^®^ Windows 10 (64-bit); and the programming language was PythonTM3.9 based on the Tensor Flow-GPU 2.6.0 deep learning framework.

#### 4.1.2. Data Preprocessing

Different operation settings may lead to different sensor values, and the obtained data represent different physical characteristics. Therefore, in order to eliminate the impact of data irregularities on the prediction effect, data normalization is carried out before the model is trained and tested, and the minimum and maximum values of the data set are converted to the range of [0,1] to improve the calculation speed of the model. The calculation formula is as follows:

(5) $${\hat{X}}_{i,j}\left(t\right)=\frac{X_{i,j}\left(t\right)-X_{min}^{j}}{X_{max}^{j}-X_{min}^{j}}$$

where, ${\hat{X}}_{i,j}\left(t\right)$ is the value obtained from the normalization of $X_{i,j}\left(t\right)$ data, and $X_{i,j}\left(t\right)$ is the jth sensor data at the ith data point at time t. $X_{min}^{j}$ and $X_{max}^{j}$ are respectively the minimum and maximum values of the data collected by the jth sensor. After the normalization of the PHM2012 data set, the original data are segmented according to the sampling points, and the input data of the network model are shown in Table 3.

#### 4.1.3. Evaluation Indicators

To extract the degradation features from the network model better, the horizontal and vertical vibration signals were used as the network input data, 0.1 s of were divided into a sample (i.e., input data were shaped $X\in R^{2560\times 2}$). The percentage of remaining service life during degradation was calculated by:

(6) $$RUL=\frac{y_{t}}{y_{i}}\times 100\%$$

where $y_{i}$ is the RUL of the total time step, and $y_{i}$ is the real RUL when the time step is t. 

The performance of the prediction results could be evaluated by a variety of indicators. To evaluate the prediction effect of the AS-TCN model, this paper used the mean absolute error (MAE) and the root mean square error (RMSE) as indicators, which were respectively calculated by:

(7) $$MAE=\frac{1}{n}\overset{n}{\underset{i=1}{\sum }}\left|y_{i}-\hat{y_{i}}\right|$$

(8) $$RMSE=\sqrt{\frac{1}{n}\overset{n}{\underset{i=1}{\sum }}{\left(y_{i}-\hat{y_{i}}\right)}^{2}}$$

where $\hat{y_{i}}$ represents the prediction result at time i, $y_{i}\;$represents the real value at time i and n is the number of samples in the test set. The smaller the absolute value of the prediction error was, the lower the MAE and RMSE values were.

#### 4.1.4. Results Analysis

The ablation study was conducted to verify the effectiveness of the sub-network of the AS-TCN model. To examine the effects of the structural parameters of the proposed model on the overall model performance, in the analysis, the key parameters of the AS-TCN were modified, either replaced or deleted, while the other parameters were kept unchanged. To further illustrate the advantages of the proposed technology, other advanced RUL prediction technologies based on deep learning were added for comparison. Finally, five methods were designed as follows: 

(1) CNN. The conventional CNN model had no DCC and adaptive shrinkage processing mechanism.

(2) AS-CNN. In this model, compared to the AS-TCN, only the TCN was replaced with the traditional CNN, while the rest of the network parameters were kept the same. 

(3) TCN. The AS-TCN is converted to TCN. The AS subnet is removed, and other structural parameters remain unchanged. This model is used to evaluate the effectiveness of the AS subnet. 

(4) DSCN [33]. A new deep separable convolutional network (DSCN) of deep prediction network is proposed, which is good to get rid of manual feature selection and learn the RUL of degradation state prediction machine from the monitoring data.

(5) CNN BiGRU [34].The most advanced method proposed by Shang et al. [34]. 

We used cross validation to deeply evaluate the performance of AS-TCN and other methods in RUL prediction. We divided the 14 data sets listed in Table 2 under two different working conditions into two groups. The first group (i.e., the seven data sets under the working condition one) used six data sets for model training, and used one of the remaining data sets as a test data set to generate RUL prediction in the corresponding direction. For example, six data sets other than the bearing 1-1 data set are used as the training set for the first cross validation, and the bearing 1-1 data set is used as the test set to generate RUL prediction of bearing 1-1, and so on.

The experimental results are shown in Table 4 and Table 5. In Table 4, we compared the MAE and RMSE of six different models. It can be seen that most of the proposed AS-TCN methods are superior to other methods. Compared with the first three models designed for ablation experiment, it is well verified that the AS subnet can improve the accuracy of RUL prediction and TCN has a good ability to capture long time series. Table 5 shows the mean values of the MAE and RMSE of several different methods. It can be seen that compared with DSCN [33], MAE and RMSE decreased by 52% and 64%, respectively, and RMSE decreased by 28% compared with CNN-BiGRU [34]. The smaller the MAE and MRSE values, the more accurate the prediction results are. The results show that AS-TCN is superior to other techniques in bearing RUL prediction.

**Figure 11:**
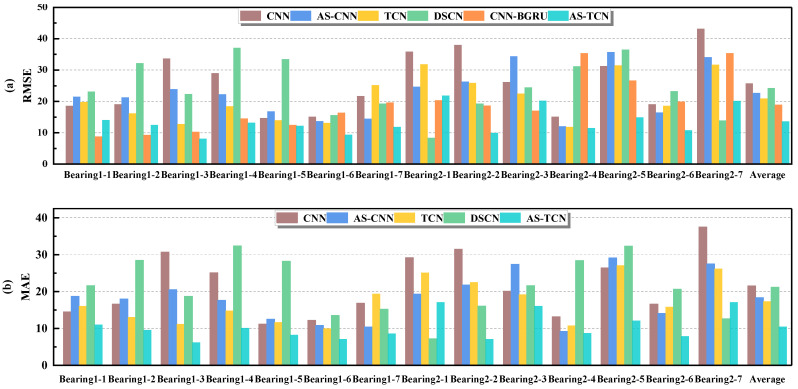
The MAE and RMSE results of the six models. (**a**) RMSE. (**b**) MAE.

**Figure 12:**
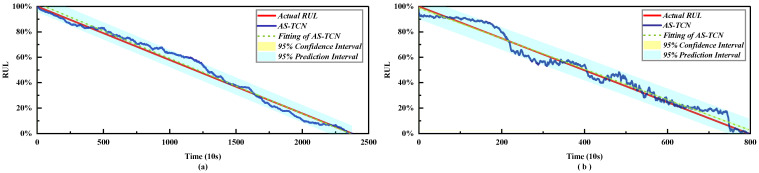
RUL prediction results of the AS-TCN. (**a**) Bearing B1–3. (**b**) Bearing B2–2.

In order to more intuitively represent the MAE and RMSE of different methods, the results in Table 4 are drawn as shown in Figure 11. The mean values of MAE and RMSE in Figure 11 are smaller than those of other methods. For MAE, in all 14 experiments, except for test bearing 2-1 and test bearing 2-7, it was significantly lower than other methods. Figure 12 shows the RUL prediction curves of bearings 1-3 and 2-2. It is assumed that the actual RUL defined by linear degradation is not exactly the same as the predicted RUL results, but the technology proposed in this study can capture the degradation trend of the bearings. Additionally, the predicted RUL result is within the 95% prediction interval of the real RUL prediction fitting line. In conclusion, the AS-TCN method can effectively predict RUL.

### 4.2. Case Study 2: XJTU-SY Bearing Dataset

#### 4.2.1. Dataset Introduction

**Figure 13:**
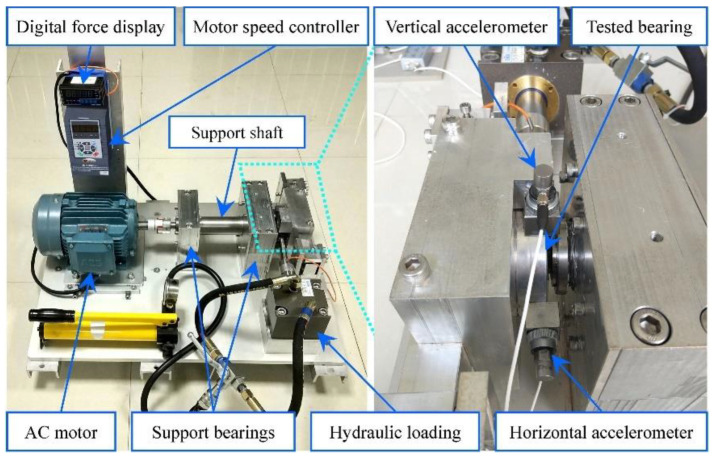
The image of the XJTU-SY test bench.

**Figure 14:**
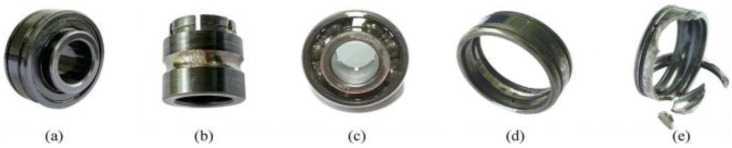
Photos of normal and degraded bearings. (**a**) Normal bearing; (**b**) inner ring wear; (**c**) roller fracture; (**d**) outer ring wear; (**e**) outer ring fracture.

**Figure 15:**
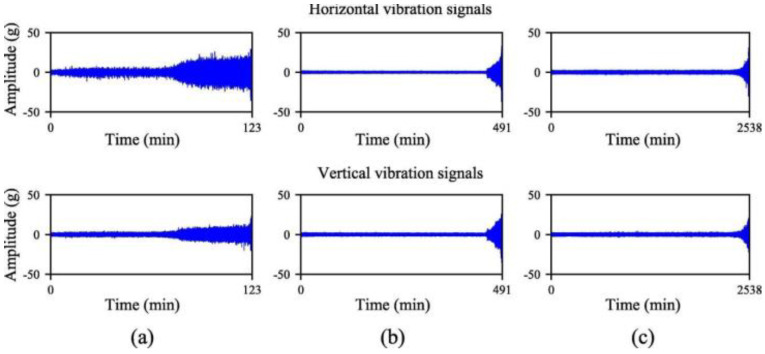
The full-life data of bearings (**a**) Bearing 1-1. (**b**) Bearing 2-1. (**c**) Bearing 2-3.

The XJTU-SY dataset was provided by Xi’an Jiaotong University and Changxing Sumiao Technology Company [35]. The test bench is shown in Figure 13. The tests were performed at two rotational speeds using LDK UER204 rolling bearings. The proposed prognostic model was validated using the operation-to-failure data under different operating conditions, as shown in Table 6. The run-to-fail vibration acceleration data were acquired by the accelerometer on the bearing housing, and it included 32,768 data samples; in 1.28 s, they were collected every 1 min with a sampling rate of 25.6 kHz. In this experiment, horizontal and vertical vibration data were used for prognosis. For safety, when the vibration amplitude exceeded 20 g, the accelerated degradation bearing test was stopped, and the corresponding time was regarded as a failure time of the bearing. A photo of the failed bearing is displayed in Figure 14. The horizontal and vertical vibration signals under two different operating conditions are presented in Figure 15. Similar to the PHM2012 dataset, horizontal and vertical vibration signals were used, and the input data is shown in Table 7. Data corresponding to the period of 1.28 s were divided into a sample; input data were $X\in {\mathbb{R}}^{\;32768\times 2}$, and the percentage of the output labeled RUL prediction result is realized by Equation (6).

#### 4.2.2. Results Comparison

As in Experiment Case I, we used cross validation to deeply evaluate the RUL prediction performance of AS-TCN and other methods. The data set division and experimental results are shown in Table 8. From Table 8, we can see that AS-TCN is superior to other methods in most bearing tests. Table 9 is the mean value of MAE and RMSE of several different methods. It can be seen that, compared with the DSCN model [33], MAE and RMSE values decreased by 48% and 42%, respectively. The results show that AS-TCN is superior to other technologies in bearing RUL prediction.

**Figure 16:**
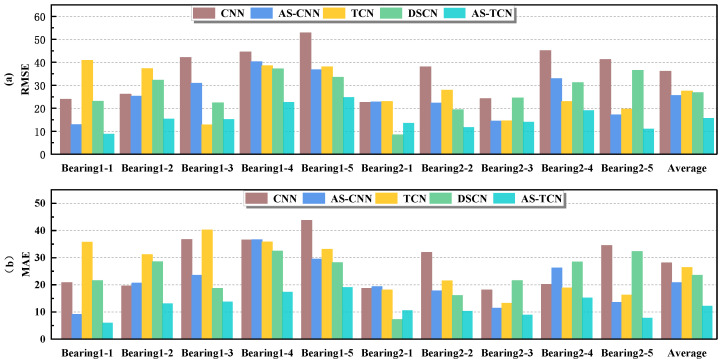
The MAE and RMSE comparison of the five different networks. (**a**) RMSE. (**b**) MAE.

**Figure 17:**
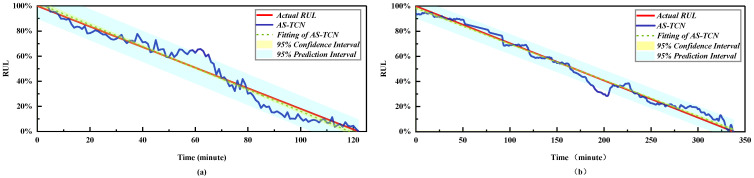
RUL prediction results of the AS-TCN. (**a**) Bearing B1–1. (**b**) Bearing B2–5.

The results in Table 8 and Table 9 are more intuitively expressed, as shown in Figure 16. The average values of MAE and RMSE in Figure 12 are smaller than other methods. For MAE and RMSE, in all 10 experiments, except for testing bearing 2-1, other methods are significantly lower than other methods. Therefore, AS-TCN has more advantages in RUL prediction. Figure 17 shows the RUL prediction curves of tested bearings 1-1 and 2-5. The predicted RUL results are within 95% of the prediction interval of the real RUL prediction fitting line, which proves that AS-TCN can capture the degradation trend of bearings well. In addition, the AS-TCN method is verified on two different datasets, which shows that the model has a good generalization ability.

## 5. Conclusions

In the process involved in the prognosis and health management framework, the prediction of remaining useful life (RUL) is a key task of PHM and is also the basis for decision-making on management activities. Therefore, this paper proposes a RUL prediction model for AS-TCN equipment to predict the RUL of rolling bearings. In addition, ablation experiments and comparisons with state-of-the-art models are conducted, and two experimental cases are provided to verify the superiority of the proposed prediction method to the other methods.

Based on the results, the following conclusions can be obtained:

(1) Adding a DCC hierarchy with a residual block to the TCN module can be used to seize longer-time collection records.

(2) The training subnet adopts an adaptive mechanism to learn the soft-thresholding function adaptively so as to reduce the information related to noise, retain degenerated features and extract the health status information of equipment.

(3) The validity of the proposed RUL prediction method based on the AS-TCN is verified by applying the rolling bearing to the test bench; the proposed method is compared with several different methods. The experimental results show that the proposed AS-TCN has excellent RUL prediction ability and thus has an important reference value for the actual remaining life prediction.

In a future study, we will consider the degradation characteristics of the same bearing under different working conditions, realize the RUL prediction of bearings under different working conditions and adaptively divide the health state and degradation state mechanism to achieve more accurate RUL prediction.

## Figures and Tables

**Table 1:** Structural parameters of the AS-TCN.

Layer	Parameter
Conv 1D	Kernel length = 12, channel = 16, stride = 4
Maxpooling 1D	Kernel length = 4, channel = 16, pooling size = 4
Dropout	Dropout rate = 0.3
AS-TCN Block 1	Kernel length = 3, channel = 12, dilation rate = 1, leaky rate = 0.2, dropout rate = 0.3
AS-TCN Block 2	Kernel length = 3, channel = 6, dilation rate = 2, leaky rate = 0.2, dropout rate = 0.3
AS-TCN Block 3	Kernel length = 3, channel = 4, dilation rate = 4, leaky rate = 0.2, dropout rate = 0.3
Dense	channel = 1

**Table 2:** Full-life table of rolling bearings under different working conditions.

Operating Conditions	Working Load	Rotating Speed (r/min)	Bearing Number	Actual Life (s)
Working condition one	4000 N	1800	Bearing1-1	28,030
			Bearing1-2	8710
			Bearing1-3	23,750
			Bearing1-4	14,280
			Bearing1-5	24,630
			Bearing1-6	24,480
			Bearing1-7	22,590
Working condition two	4200 N	1650	Bearing2-1	9110
			Bearing2-2	7970
			Bearing2-3	19,550
			Bearing2-4	7510
			Bearing2-5	23,110
			Bearing2-6	7010
			Bearing2-7	2300

**Table 3:** The input data after data preprocessing of PHM2012 dataset.

Bearing Number	[Samples, Samples Point, Channel]	Bearing Number	[Samples, Samples Point, Channel]
Bearing1-1	[2803, 2560, 2]	Bearing2-1	[911, 2560, 2]
Bearing1-2	[871, 2560, 2]	Bearing2-2	[797, 2560, 2]
Bearing1-3	[2375, 2560, 2]	Bearing2-3	[1955, 2560, 2]
Bearing1-4	[1428, 2560, 2]	Bearing2-4	[751, 2560, 2]
Bearing1-5	[2463, 2560, 2]	Bearing2-5	[2311, 2560, 2]
Bearing1-6	[2448, 2560, 2]	Bearing2-6	[701, 2560, 2]
Bearing1-7	[2259, 2560, 2]	Bearing2-7	[2300, 2560, 2]

**Table 4:** The evaluation indicators of the AS-TCN model and several comparison models.

Test Bearing	Training Bearings	CNN		AS-CNN		TCN		DSCN [33]		CNN-BGRU [34]	AS-TCN	
		MAE	RMSE	MAE	RMSE	MAE	RMSE	MAE	RMSE	RMSE	MAE	RMSE
Bearing 1-1	B1-2~B1-7	14.5	18.6	18.7	21.5	16.1	19.8	21.6	23.1	8.8	11	14
Bearing 1-2	B1-1,B1-3~B1-7	16.6	19.1	18	21.3	13.1	16.2	28.5	32.2	9.3	9.6	12.5
Bearing 1-3	B1-1~B1-2,B1-4~B1-7	30.7	33.7	20.5	23.9	11.2	12.8	18.7	22.4	10.3	6.2	8.1
Bearing 1-4	B1-1~B1-3,B1-5~B1-7	25.1	29	17.6	22.3	14.8	18.5	32.4	37.1	14.6	10.1	13.2
Bearing 1-5	B1-1~B1-4,B1-6~B1-7	11.2	14.7	12.5	16.8	11.7	14	28.2	33.5	12.5	8.2	12.2
Bearing 1-6	B1-1~B1-5,B1-7	12.2	15.1	10.8	13.7	10	13.1	13.5	15.6	16.4	7.1	9.4
Bearing 1-7	B1-1~B1-6	16.8	21.7	10.4	14.5	19.4	25.2	15.2	19.3	19.7	8.6	11.8
Bearing 2-1	B2-2~B2-7	29.2	35.9	19.3	24.7	25.1	31.9	7.2	8.4	20.4	17.1	21.8
Bearing 2-2	B2-1,B2-3~B2-7	31.5	38	21.8	26.3	22.5	25.9	16.1	19.3	18.7	7.1	9.9
Bearing 2-3	B2-1~B2-2,B2-4~B2-7	20.1	26.2	27.4	34.4	19.2	22.5	21.6	24.5	17	16.1	20.2
Bearing 2-4	B2-1~B2-3,B2-5~B2-7	13.2	15.1	9.2	12.1	10.8	11.9	28.4	31.2	35.4	8.7	11.5
Bearing 2-5	B2-1~B2-4,B2-6~B2-7	26.4	31.3	29.1	35.8	27.1	31.5	32.3	36.5	26.7	12.1	14.9
Bearing 2-6	B2-1~B2-5,B2-7	16.6	19.1	14.1	16.5	15.8	18.6	20.6	23.3	20	7.9	10.8
Bearing 2-7	B2-1~B2-6	37.5	43.2	27.5	34.1	26.2	31.7	12.6	13.9	35.4	17.1	20.1

**Table 5:** Comparison of the average results of different methods under the PHM2012 dataset.

Method	RMSE Value	Difference	MAE Value	Difference
CNN	25.8	↑12.2	21.5	↑11.5
AS-CNN	22.7	↑8.1	18.4	↑8.4
TCN	21	↑7.4	17.4	↑7.4
DSCN [33]	24.3	↑10.7	21.2	↑11.2
CNN-BGUR [34]	18.9	↑5.3	–	–
**AS-TCN**	**13.6**	–	**10**	–

**Table 6:** Full-life results of rolling bearings under different working conditions.

Operating Conditions	Working Load	Rotating Speed (r/min)	Bearing Number	Actual Life
Working condition one	12,000 N	2100	Bearing 1-1	2 h and 3 min
			Bearing 1-2	2 h and 41 min
			Bearing 1-3	2 h and 38 min
			Bearing 1-4	2 h and 2 min
			Bearing 1-5	52 min
Working condition two	11,000 N	2250	Bearing 2-1	8 h and 11 min
			Bearing 2-2	2 h and 41 min
			Bearing 2-3	8 h and 53 min
			Bearing 2-4	42 min
			Bearing 2-5	5 h and 39 min

**Table 7:** The input data after data preprocessing of XJTU-SY dataset.

Bearing Number	[Samples, Samples Point, Channel]	Bearing Number	[Samples, Samples Point, Channel]
Bearing 1-1	[123, 32768, 2]	Bearing 2-1	[491, 32768, 2]
Bearing 1-2	[161, 32768, 2]	Bearing 2-2	[161, 32768, 2]
Bearing 1-3	[158, 32768, 2]	Bearing 2-3	[533, 32768, 2]
Bearing 1-4	[122, 32768, 2]	Bearing 2-4	[42, 32768, 2]
Bearing 1-5	[52, 32768, 2]	Bearing 2-5	[339, 32768, 2]

**Table 8:** The evaluation indicators of the AS-TCN network and several other networks.

Test Bearing	Training Bearings	CNN		AS-CNN		TCN		DSCN [33]		AS-TCN	
		MAE	RMSE	MAE	RMSE	MAE	RMSE	MAE	RMSE	MAE	RMSE
Bearing 1-1	B1-2~B1-5	20.8	23.9	9.1	12.9	35.7	40.8	21.6	23.1	6.0	8.8
Bearing 1-2	B1-1,B1-3~B1-5	19.6	26.1	20.7	25.3	31.1	37.2	28.5	32.2	13.2	15.4
Bearing 1-3	B1-1~B1-2,B1-4~B1-5	36.7	42.1	23.5	30.9	40.2	12.8	18.7	22.4	13.8	15.2
Bearing 1-4	B1-1~B1-3,B1-5	36.5	44.5	36.6	40.3	35.8	38.5	32.4	37.1	17.4	22.7
Bearing 1-5	B1-1~B1-4	43.7	52.8	29.5	36.8	33.1	38	28.2	33.5	19.1	24.8
Bearing 2-1	B2-2~B1-5	18.7	22.6	19.3	22.7	18.1	22.9	7.2	8.4	10.6	13.6
Bearing 2-2	B2-1,B2-3~B2-5	31.9	38	17.8	22.3	21.5	27.9	16.1	19.3	10.3	11.8
Bearing 2-3	B2-1~B2-2,B2-4~B2-5	18.1	24.2	11.4	14.4	13.2	14.5	21.6	24.5	9.0	14.1
Bearing 2-4	B2-1~B2-3,B2-5	20.2	45.1	26.2	32.9	18.8	22.9	28.4	31.2	15.3	19.1
Bearing 2-5	B2-1~B2-4	34.5	41.2	13.5	17.1	16.2	19.7	32.3	36.5	7.8	11.1

**Table 9:** Comparison of the average results of different methods under the XJTU-SY dataset.

Method	RMSE Value	Difference	MAE Value	Difference
CNN	36.1	↑20.5	28.1	↑15.9
AS-CNN	25.6	↑10.0	21.1	↑8.9
TCN	27.5	↑11.9	25.3	↑13.1
DSCN	26.8	↑11.2	23.5	↑11.3
**AS-TCN**	**15.6**	–	**12.2**	–

## Data Availability

The data used to support this study are available at https://biaowang.tech/xjtu-sy-bearing-datasets/ (accessed on 27 January 2021).
